# The top-scoring ‘N’ algorithm: a generalized relative expression classification method from small numbers of biomolecules

**DOI:** 10.1186/1471-2105-13-227

**Published:** 2012-09-11

**Authors:** Andrew T Magis, Nathan D Price

**Affiliations:** 1 Institute for Systems Biology, 401 Terry Ave N, Seattle, WA, 98109, USA; 2 Center for Biophysics and Computational Biology, University of Illinois, Urbana, IL, 61801, USA

**Keywords:** Classification, Top-scoring pair, Relative expression, Cross validation, Support vector machine, Graphics processing unit, Microarray

## Abstract

**Background:**

Relative expression algorithms such as the top-scoring pair (TSP) and the top-scoring triplet (TST) have several strengths that distinguish them from other classification methods, including resistance to overfitting, invariance to most data normalization methods, and biological interpretability. The top-scoring ‘N’ (TSN) algorithm is a generalized form of other relative expression algorithms which uses generic permutations and a dynamic classifier size to control both the permutation and combination space available for classification.

**Results:**

TSN was tested on nine cancer datasets, showing statistically significant differences in classification accuracy between different classifier sizes (choices of *N*). TSN also performed competitively against a wide variety of different classification methods, including artificial neural networks, classification trees, discriminant analysis, k-Nearest neighbor, naïve Bayes, and support vector machines, when tested on the Microarray Quality Control II datasets. Furthermore, TSN exhibits low levels of overfitting on training data compared to other methods, giving confidence that results obtained during cross validation will be more generally applicable to external validation sets.

**Conclusions:**

TSN preserves the strengths of other relative expression algorithms while allowing a much larger permutation and combination space to be explored, potentially improving classification accuracies when fewer numbers of measured features are available.

## Background

Relative expression algorithms such as the top-scoring pair (TSP) [1] and the top-scoring triplet (TST) [2] represent powerful methods for disease classification, primarily focused on the creation of simple, yet effective classifiers. These algorithms have several strengths that distinguish them from other classification methods. First, only the ranks of the expression data are used, rather than the expression values directly, therefore these algorithms are invariant to data normalization methods that preserve rank-order. For example, quantile normalization is a rank-preserving common practice in microarray analysis to remove technical sources of variance between arrays [3]. It is therefore preferable that the classification algorithm be insensitive to such normalization procedures, particularly in meta-analyses combining data from multiple studies or in a clinical setting where additional measurements beyond the features used to build the classifier would be needed to apply the normalization step. Second, relative expression classifiers make use of only a few features to build each classifier, and require relatively little to no parameter tuning. As a result, the algorithms are generally resistant to overfitting, in which an algorithm learns to classify the noise of the training set rather than the true phenotypic signal of interest. Moreover, the small number of features in relative expression algorithms lends itself well to the development of inexpensive clinical tests [4]. Third, an underappreciated aspect of relative expression algorithms involves their potential for biological interpretation. The simplicity of these algorithms, in which the ranks of a few features shift positions in a predictable way between two phenotypic classes, suggests that the features participating in a highly accurate classifier may represent or reflect an underlying biological role for those features in the phenotypes being classified. Relative expression algorithms may therefore serve as hypothesis generators for additional study. This characteristic may become particularly relevant as classification methods move increasingly more into technologies such as secretomics and miRNA expression measurements that, at present, result in fewer measurements per sample than do transcriptomes.

In this paper we present a new formulation of the relative expression classification algorithm that generalizes the idea of pairwise rank comparisons (TSP) and triplet rank comparisons (TST) into generic permutation rank comparisons, where the size of the classifier is not defined *a priori*. This algorithm is called the top-scoring ‘*N*’ (TSN), where *N* is a variable indicating the size of the classifier. As such, TSP and TST can be thought of as special cases of the general TSN algorithm (just with a fixed *N* = 2 or *N* = 3, respectively). Because the classifier size is unconstrained, TSN can explore a much larger permutation and combination space than that available to either TSP or TST. All of the results presented in this paper used *no more than sixteen features* from any of the training sets.

The classification accuracy of the existing relative expression algorithms has been demonstrated in several studies. Classifiers identified using relative expression algorithms have been used to distinguish multiple cancer types from normal tissue based on expression data [1,2,4,5] as well as to predict cancer outcomes and model disease progression [6]. Furthermore, relative expression algorithms perform competitively when compared to other, often more complex, classification methods, including support vector machines [7], decision trees [8] and neural networks [9]. Relative expression algorithms have also been applied in a network context, illustrating the dysregulation of cellular pathways in disease phenotypes [10].

We first demonstrate that both TSP and TST are special cases of the TSN algorithm. We illustrate the performance of a range of TSN classifier sizes on a set of nine cancer datasets. Finally, we demonstrate that TSN performs competitively when compared to a broad range of classification models, including artificial neural networks, classification trees, and support vector machines, using data and results from the FDA-sponsored Microarray Quality Control II project (MAQC-II) [11].

## Methods

### Overview of relative expression algorithms TSP and TST

Given two classes of samples *C* = {*C*_*1*_*, C*_*2*_}, for which ranked expression data are available on *M* features *X* = {*x*_*1*_,…,*x*_*M*_}, the TSP algorithm [1] searches for the feature pair {*x*_*i*_*, x*_*j*_} that maximizes the TSP score Δ_*i,j*_, defined as:

$$\Delta_{i,j}=|\mathrm{Pr}\left(x_{i}<x_{j}|C=C_{1}\right)-Pr\left(x_{i}<x_{j}|C=C_{2}\right)|,i\neq j$$

The TSP algorithm identifies the best pair of features for which the rank of *x*_*i*_ falls lower than the rank of *x*_*j*_ in most or all samples in class *C*_*1*_, and the rank of *x*_*i*_ falls lower than the rank of *x*_*j*_ in few or no samples of class *C*_*2*_. The max (Δ_*i,j*_ = 1) indicates a perfect classifier on the training set in which no samples deviate from this pattern. Classification is performed by comparing the ordering of features {*x*_*i*_*, x*_*j*_} in each sample of the test set to the orderings associated with the two classes. A variant on this algorithm known as k-TSP makes use of multiple disjoint pairs to improve classification accuracy [5].

The top-scoring triplet (TST) algorithm [2] extends the TSP algorithm to triplets of features. The six unique permutations π_1_,…,π_6_ of each feature triplet {*x*_*i*_*, x*_*j*_*, x*_*k,*_} are now considered explicitly, where:

$$\begin{array}{l} \pi_{1}=\left\{x_{i}<x_{j}<x_{k}\right\},\pi_{2}=\left\{x_{i}<x_{k}<x_{j}\right\},\pi_{3}=\left\{x_{j}<x_{i}<x_{k}\right\}\\ \pi_{4}=\left\{x_{j}<x_{k}<x_{i}\right\},\pi_{5}=\left\{x_{k}<x_{i}<x_{j}\right\},\pi_{6}=\left\{x_{k}<x_{j}<x_{i}\right\} \end{array}$$

These permutation counts are accumulated for each sample of the training set, and the TST score Δ_*i,j,k*_ to be maximized is then calculated as follows:

$$\Delta_{i,j,k}=\frac{1}{2}\sum_{m=1}^{6}|Pr\left(\pi_{m}|C=C_{1}\right)-Pr\left(\pi_{m}|C=C_{2}\right)|,i\neq j\neq k$$

### The top-scoring N algorithm

**Figure 1:**
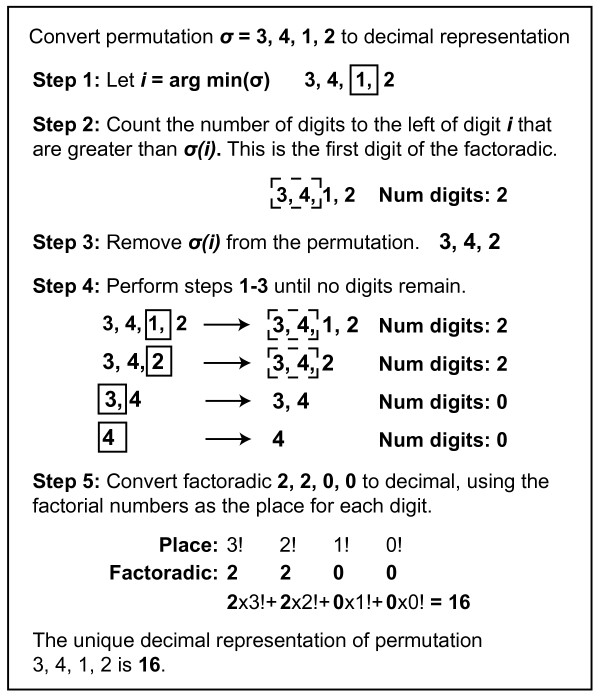
**The Lehmer code.** A complete translation from permutation to decimal, by way of the factoradic, for a permutation of size 4. Each permutation is mapped to a single unique decimal representation. Two additional translations from permutation to factoradic are shown in Additional file 1: Figure S2.

The top-scoring ‘N’ algorithm, as the name implies, extends these relative expression algorithms to a generic permutation size. Within the context of feature permutations, TSP and TST can be thought of as special cases of the TSN algorithm, where a fixed *N* = 2 and *N* = 3 are used, respectively. The TSN algorithm uses a nonstandard counting system known as factoradics, or factorial-radix numbers. Briefly, factoradics can be described as a mixed-radix counting system in which the multiplicative factor for each digit placeholder is derived from the set of factorial numbers. An example of factoradics compared to two other common fixed-radix counting systems is shown in Additional file 1: Figure S1. Given that the factoradic counting system is intimately related to the factorial numbers, it is perhaps not surprising that there is a relationship between factoradics and permutations. There exist *N*! permutations of a set of *N* objects, and therefore each permutation of *N* objects may be represented by an integer from 0 to *N*!-1. Factoradics provide a mechanism by which permutations may be uniquely represented, and the translation between a permutation and its corresponding factoradic is known as the Lehmer code (Figure 1). Using factoradics, every permutation has a one-to-one correspondence with a decimal number. Several examples of permutation-to-decimal translations via factoradics are shown in Additional file 1: Figure S2.

The TSN algorithm works as follows: given two classes of samples *C* = {*C*_*1*_, *C*_*2*_} with rank values for *M* features {*x*_*1*_,…,*x*_*M*_}, and a classifier size *N,* the TSN algorithm identifies the feature set *X* = {*x*_*i*_,*x*_*j*_,…*x*_*N*_} that maximizes the sum of the difference of the permutation probability distribution between the two classes:

$$\Delta_{X}=\frac{1}{2}\sum_{m=1}^{N!}|Pr\left(\sigma_{m}|C=C_{1}\right)-Pr\left(\sigma_{m}|C=C_{2}\right)|$$

where *σ*_*m*_ is the *m*th permutation of the classifier *X*. Recall that there are *N*! possible permutations of *X.* The permutation probability distribution for each class is determined by mapping the permutation of *X* for each training set sample to its corresponding factoradic, converting the factoradic to decimal representation, and using this as an index into a histogram of size *N*!. Once normalized by the number of samples in each class, the histogram represents the permutation probability distribution for that feature set on that training set class. When the two histograms are completely disjoint (i.e., there are no overlapping permutations between the two classes), the TSN score Δ_*X*_ = 1.

In addition to the primary TSN score, a secondary score γ is calculated in the event of ties between two classifiers. This is simply the distance in rank between the first and last element of the classifier *X* for each sample, summed over all the samples of the training set:

$$\gamma_{X}=\sum_{i=1}^{s}\left|R_{X\left(N\right),i}-R_{X\left(1\right),i}\right|$$

where *S* is equal to the number of samples in the training set and *N* the size of the classifier *X*. *R* refers to the rank, and *X*(1) and *X*(*N*) are the first and last elements of the classifier, respectively. In the case of ties in the primary TSN score, the classifier chosen will have the largest distance in rank between the upper and lower elements of the classifier.

In the case where *N* = 2, the TSN algorithm simply reduces to the TSP algorithm, since *X*_*2*_ = {*x*_*i*_*, x*_*j*_}, and *Pr*(σ_1_) = *Pr*(*x*_*i*_ < *x*_*j*_). In the case where *N* = 3, the TSN algorithm reduces to the TST algorithm, since *X*_*3*_ = {*x*_*i*_*,x*_*j*_*,x*_*k*_} and *Pr*(σ_*m*_) = *Pr*(*π*_*m*_). Because the TSN algorithm uses factoradics to uniquely represent any permutation of any size classifier, it allows TSP and TSP classifiers to be used in concert as well as allowing for even larger classifiers to be explored.

The choice of *N* is clearly important in the determination of a new classifier for a training set. The simplest method is to choose the value of *N* with the greatest classification accuracy after iteration over a range of *N*. This method would reveal the apparently most effective classifier size. In this case the experimenter is artificially choosing the ‘best’ value of *N* for a given dataset. However, in fair comparisons with other classification methods it is important that the choice of *N* not be made *a posteriori* (once the best classifier and value of *N* have been determined) to avoid overly optimistic error estimates. We do not choose the value of *N outside* the cross validation loop, but rather dynamically select the value of *N at each iteration* of the cross validation loop; the choice is made based on the apparent accuracy of that value of *N* on the training set. We call this version of the algorithm *dynamic N*. Apparent accuracy is calculated by first finding the highest scoring classifier on the training set for each value of *N* in a range specified by the user. The value of *N* with the highest apparent accuracy on the training set is then applied to the test set. In the case of ties in apparent accuracy for multiple values of *N*, the algorithm chooses the smallest tied value of *N* for the classifier at that iteration of the cross validation loop. This process is repeated at each iteration of the cross validation loop. Note that this method does not preclude the user from artificially choosing the best value of *N* (outside of cross validation) for other purposes, but is rather a mechanism to avoid bias during cross validation. This allows us to make fair comparisons of the TSN algorithm with other classification methods without potentially biasing the results in our favor.

### Classification with TSN

**Figure 2:**
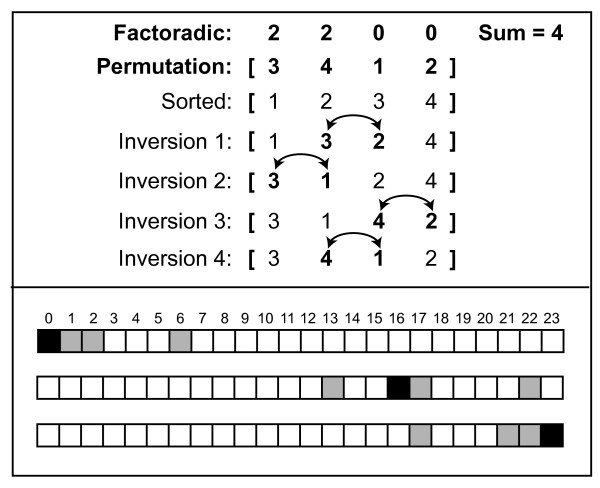
**Inversions.** (**Top**) There are four inversions required to translate the sorted list [1 2 3 4] into the permutation [3 4 1 2]. The sum of the digits of the factoradic give the number of inversions required to translate one permutation into another. (**Bottom**) The grey squares indicate the set of permutations that have a single inversion distance from the original (black) permutations.

Once the highest scoring classifier *X* is identified using a training set, prediction on a test set is performed by comparing the classifier permutation for each sample of the test set to the permutation probability distribution of the classifier for each class. A class is predicted for each sample based on which permutation probability is higher for the permutation of that sample. For example, given a classifier size of *N* = 4, if a particular sample in the test set contains permutation 16, that sample is classified as class 1 or class 2 based on which class has higher permutation probability for permutation 16 in the training set. A special case may occur during classification, where the probability for a test set permutation is equal (or zero) for both classes. In this case, the algorithm adopts a maximum likelihood approach to classify the sample. First, all permutations are identified with an inversion distance of 1 from the original permutation. The inversion distance is defined as the number of adjacent swaps required to convert one permutation into another (Figure 2, top panel). For example, given a classifier of size 4, each permutation has a set of three permutations with an inversion distance of 1. For permutation 16, this set includes permutations 13, 17, and 22 (Figure 2, bottom panel). Once the single-inversion permutation set is identified, the permutation probability for this set is summed for each class. The class with the higher probability is chosen. If the single-inversion distance sums are the same between the two classes, the algorithm repeats the calculation for the permutations with inversion distance 2, and so on. If a choice cannot be made, which only occurs if both classes have identical permutation probability distributions, that sample is considered an incorrect prediction for that iteration of the cross validation loop.

### Implementation of TSN

While the TSN algorithm can theoretically explore a very large permutation space, the computational requirements of the algorithm rise very quickly and to avoid overfitting the number of permutations explored must be scaled to what is reasonable given available sample numbers. The complexity of TSN is ONMN!, where *M* is the number of features and *N* is the size of the classifier. We have previously shown [12] that the graphics processing unit (GPU) is highly efficient when applied to easily parallelizable algorithms such as TSP and TST. Given that TSN preserves the parallel nature of the other relative expression algorithms, it is also easily applied to the GPU. However, given that GPU hardware is not yet widely available to many researchers, we are releasing the source code for both GPU and CPU implementations of the TSN algorithm. TSN has been implemented for both the GPU and the CPU in the MATLAB computing environment.

**Figure 3:**
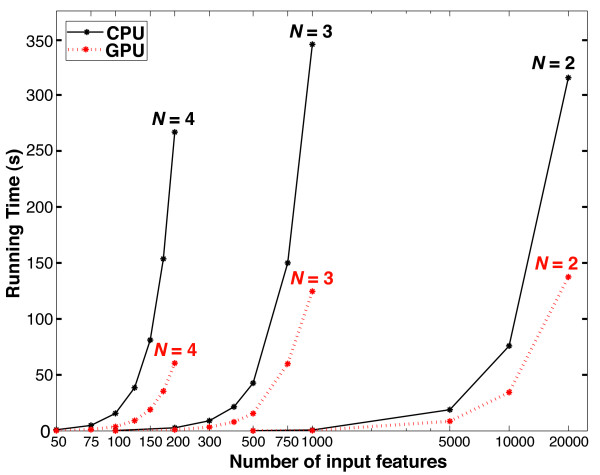
**GPU *vs. *CPU running times.** Running times for *N* = 2, *N* = 3, and *N* = 4 over a range of input feature sizes. Each point is the mean of three independent runs of the software. The CPU running time for *N* = 2 over 20,000 features is similar to the running times for *N* = 3 over 1000 features and *N* = 4 over 200 features. The CPU version of TSN was run on a single core of a 2.4 GHz Intel Core 2 processor. The GPU version of TSN was run on an NVIDIA Tesla C2050. The speedup due to the GPU improves as the value of *N* gets higher: for *N* = 2, the speedup is 2.3X, for *N* = 3 the speedup is 2.8X, and for *N* = 4 the speedup is 4.4X. Running times reflect a single iteration of the algorithm and do not include multiple iterations such as cross validation. Note that running times are also a function of the number of samples in the dataset; there were 70 samples in this dataset.

The GPU is a specialized hardware device normally used in graphics rendering. The nature of graphics rendering involves large numbers of vector and matrix operations performed in real-time, thus the GPU architecture emphasizes massive parallelism. Driven by the billion-dollar gaming industry, the GPU has developed into a powerful tool currently able to reach over 1 TFLOP (trillion floating point operations per second) on a single chip in single precision operations. With NVIDIA’s release of the Compute Unified Device Architecture (CUDA) in 2007, general-purpose computation on the GPU became accessible. GPUs are increasingly being applied to computationally intensive scientific problems, including molecular dynamics simulations [13], weather prediction [14], quantum chemistry [15], bioinformatics [16], and medical imaging [17]. Plots of running times for *N* = 2, *N* = 3, and *N* = 4 over a wide range of input feature sizes are shown in Figure 3. The speedup of the GPU over the CPU implementations of TSN improves as the value of *N* gets higher, ranging from 2.3X for *N* = 2 to 4.4X for *N* = 4. Pseudocode for the operation of the core TSN algorithm is shown in Additional file 1: Figure S3. All source code for both the CPU and GPU implementations is freely available on http://price.systemsbiology.net/downloads.php.

## Results and discussion

### Multiple values of N

TSN has been tested on nine cancer datasets that were used in the previous k-TSP and TST papers [2,5] for comparison between different values of *N*. These datasets represent a wide range of cancers, including colon [18], leukemia [19], central nervous system lymphoma (CNS) [20], diffuse large B-cell lymphoma (DLBCL) [21], prostate [22-24], and a global cancer map (GCM) dataset [25]. As discussed in the methods section, the TSN algorithm can be used in two different ways: the choice of *N* can be made *a posteriori* after all fixed values have been tested, or the choice of *N* can be made at each iteration of the cross validation loop (*dynamic N)* using apparent accuracy. Apparent accuracy is calculated by first finding the highest scoring classifier on the training set for each value of *N* in a range specified by the user. The value of *N* with the highest apparent accuracy on the training set is then applied to the test set. In order to directly compare the accuracies based on the number of permutations of features, we chose 16 features for *N* = 2, 10 features for *N* = 3, and 9 features for *N* = 4. This results in approximately 120 combinations for each value of *N.* The reason for choosing different numbers of features for each value of *N* is to equalize the combination space for each classifier size. For example, a classifier of size *N =* 2 given 16 features can explore 2! = 2 permutations over $\left(\underset{2}{16}\right)=120$ combinations. A classifier of size *N =* 3 given 10 features can explore 3! = 6 permutations over $\left(\underset{3}{10}\right)=120$ combinations. A classifier of size *N* = 4 given 9 features can explore 4! = 24 permutations over $\left(\underset{4}{9}\right)=126$ combinations. As a result, any difference in accuracy between these two classifiers depends primarily on the permutation space being explored and not the combination space (which is held relatively constant). The features were chosen to be the most differentially expressed genes based on the Wilcoxon rank sum test, again selected within each iteration of the cross validation loop to avoid overly optimistic estimates.

**Figure 4:**
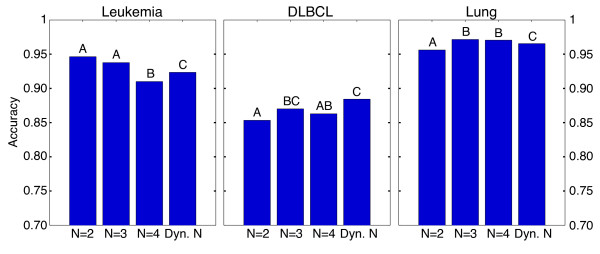
**Results of TSN classification on cancer datasets.** Results of 100 rounds of 5-fold cross validation over a range of *N* = {2,3,4} where the number of differentially expressed probes is different for each value of *N* {16,10,9}. This yields approximately the same number of possible combinations for each value of *N* (~120), illustrating how classification accuracy can be determined by the permutation itself, not just the number of combinations available. Results shown include accuracies of fixed values of *N* as well as the *dynamic N* algorithm described in the methods section. Statistical differences were calculated using the nonparametric Kruskal-Wallis one-way analysis of variance by ranks, and a p-value < 0.05 was considered significant. If bars share the same letter they are not statistically different. The datasets are derived from [2] and represent a wide range of cancers. Significance plots for all nine cancer datasets are in Additional file 1: Figure S4.

Shown in Figure 4 are the results of TSN being applied to three of the cancer datasets with fixed values of *N* as well as *dynamic N* using 5-fold cross validation. To determine statistically significant differences between values of *N*, we ran 100 iterations of 5-fold cross validation on each of the nine cancer datasets. Each iteration of cross validation randomly selected different training and test sets, allowing us to measure the distribution of accuracies for each value of *N.* This was done for fixed *N* = 2, fixed *N* = 3, fixed *N* = 4, and *dynamic N =* {2,3,4} as described above. Because the resulting distributions of accuracies failed a Kolmogorov-Smirnov normality test, we used the non-parametric Kruskal-Wallis one-way analysis of variance by ranks to measure differences between the groups. A p-value < 0.05 was considered significant. Significant differences are indicated by letters above each bar; if two bars share the same letter they are not statistically different. Significance plots for all nine cancer datasets are shown in Additional file 1: Figure S4. All raw data is included in Additional file 2.

It is clear from Figure 4 that the value of *N* can have a significant effect on the resulting accuracy of the classifier, which indicates that the larger permutation space afforded by larger values of *N* can be useful in identifying an effective classifier. In the Leukemia dataset, for example, *N* = 2 and *N* = 3 produced the apparently most effective classifiers; in the Lung dataset, *N* = 3 and *N* = 4 were the apparent best. In four of the nine datasets (DLBCL, Prostate2, Prostate3, and GCM), *dynamic N* yielded no significant difference in accuracy with the highest-scoring fixed value of *N.* In two additional datasets (Leukemia and Lung), the *dynamic N* accuracy is statistically in between the highest- and lowest-scoring values of *N.* In the remaining three datasets (Colon, CNS, and Prostate1), the *dynamic N* accuracy is not significantly different from the lowest-scoring fixed value of *N.* The *dynamic N* TSN result is the fair estimate of how well the algorithm would be expected to perform with optimization for *N*, without the bias that is introduced by choosing the apparently best *N* after the error estimate has been made.

### Microarray quality control II datasets

Published in 2010, the Microarray Quality Control II dataset (MAQC-II) [11] was produced by the National Center for Toxicological Research at the United States Food and Drug Administration in collaboration with 96 universities and companies from around the world. One goal of the project was to build a set of microarray data that could be used to validate classification methods in a rigorous and systematic manner. To this end, six different microarray datasets representing a range of phenotypes, microarray platforms, and sample sizes were selected by the consortium. Each dataset was partitioned into one or more *endpoints*, where an endpoint represents a class partition to be predicted. A total of thirteen endpoints were represented by the six datasets. Each endpoint consisted of a training set as well as an independently collected validation set. A listing of the MAQC-II datasets and endpoints used in this study is provided in Table 1. Note that only five of the datasets representing nine endpoints are currently available for public download from the Gene Expression Omnibus (GSE16716). We tested TSN on all endpoints for which data was available. Thirty-six independent groups using a variety of classification methods, including artificial neural networks, classification trees, discriminant analysis, k-Nearest neighbor, naïve Bayes, and support vector machines, analyzed the MAQC-II training sets and provided nearly 20,000 models to the MAQC-II consortium. It should be noted the groups were not restricted to a single classification method, and many chose to use different methods for the different endpoints based on what they determined would be the most successful. As a result, our single classification method, TSN, is being compared against ensembles of methods by most MAQC-II participants. Each group ultimately nominated a single model from each endpoint training set to be tested on the corresponding validation set, and these models were compiled into a list of final predictions. To further test the classification algorithms, the MAQC-II consortium swapped the training and validation sets for each endpoint, and each group submitted predictions for the swapped datasets. TSN was tested against the groups that submitted validation set predictions for every available endpoint on both original and swapped data. A listing of the participants and their respective classification methods used in this paper is provided in Table 2.

**Table 1:** The five MAQC-II datasets, representing endpoints A through I that are available from the Gene Expression Omnibus

**Dataset**	**Endpoint**	**Description**	**Platform**
**Hamner**	A	Lung tumorigen *vs.* non-tumorigen	Affymetrix Mouse 430 2.0
**Iconix**	B	Non-genotoxic liver carcinogens *vs.* non-carcinogens	Amersham Uniset Rat 1 Bioarray
**NIEHS**	C	Liver toxicants *vs.* non-toxicants	Affymetrix Rat 230 2.0
**Breast Cancer**	D	Pre-operative treatment response	Affymetrix Human U133A
	E	Estrogen receptor status	
**Multiple Myeloma**	F	Overall survival milestone outcome	Affymetrix Human U133 Plus 2.0
	G	Event-free survival milestone outcome	
	H	Gender of patient (positive control)	
	I	Random class labels (negative control)	

**Table 2:** The participants that submitted models for every endpoint (original and swap) in the MAQC-II study, and the classification methods used

**Code**	**Name**	**Classification algorithm(s) used**
**CAS**	Chinese Academy of Sciences	Naïve Bayes, Support Vector Machine
**CBC**	CapitalBio Corporation, China	k-Nearest Neighbor, Support Vector Machine
**Cornell**	Weill Medical College of Cornell University	Support Vector Machine
**FBK**	Fondazione Bruno Kessler, Italy	Discriminant Analysis, Support Vector Machine
**GeneGo**	GeneGo, Inc.	Discriminant Analysis, Random Forest
**GHI**	Golden Helix, Inc.	Classification Tree
**GSK**	GlaxoSmithKline	Naïve Bayes
**NCTR**	National Center for Toxicological Research, FDA	k-Nearest Neighbor, Naïve Bayes, Support Vector Machine
**NWU**	Northwestern University	k-Nearest Neighbor, Classification Tree, Support Vector Machine
**SAI**	Systems Analytics, Inc.	Discriminant Analysis, k-Nearest Neighbor, Machine Learning, Support Vector Machine, Logistic Regression
**SAS**	SAS Institute, Inc.	Classification Tree, Discriminant Analysis, Logistic Regression, Partial Least Squares, Support Vector Machine
**Tsinghua**	Tsinghua University, China	Classification Tree, k-Nearest Neighbor, Recursive Feature Elimination, Support Vector Machine
**UIUC**	University of Illinois, Urbana-Champaign	Classification Tree, k-Nearest Neighbor, Naïve Bayes, Support Vector Machine
**USM**	University of Southern Mississippi	Artificial Neural Network, Naïve Bayes, Sequential Minimal Optimization, Support Vector Machine
**ZJU**	Zejiang University, China	k-Nearest Neighbor, Nearest Centroid

The metric chosen by the MAQC-II consortium to rate the classification models was the Matthew’s Correlation Coefficient (MCC). The MCC has several advantages over the accuracy/sensitivity/specificity standard, as it is able to detect inverse correlations as well as being sensitive to the overall size of the training sets. MCC values range from +1 (perfect prediction) to −1 (perfect inverse prediction), with 0 indicating random prediction. Note that unbeknownst to the original study participants, endpoints H and I were replaced by a positive control (gender of the study participants) and a negative control (random class assignments), respectively. Therefore, it was expected that endpoint H would result in very high prediction MCC and endpoint I would result in MCC close to zero. The MCC is calculated as follows:

$$\mathrm{MCC}=\frac{\mathrm{TP}\times \mathrm{TN}-\mathrm{FP}\times \mathrm{FN}}{\sqrt{\left(\mathrm{TP}+\mathrm{FP}\right)\left(\mathrm{TP}+\mathrm{FN}\right)\left(\mathrm{TN}+\mathrm{FP}\right)\left(\mathrm{TN}+\mathrm{FN}\right)}}$$

If any of the sums in the denominator of the MCC are zero, the denominator is set to be one, resulting in an MCC equal to zero.

**Figure 5:**
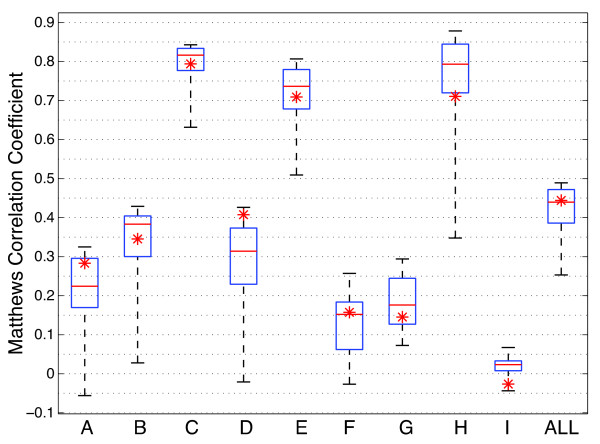
**Results of TSN classification on MAQC-II datasets.** MCC of MAQC-II endpoints A through I, based on models learned on the training set and then applied to the validation set. MCC values range from +1 (perfect prediction) to −1 (perfect inverse prediction), with 0 indicating random prediction. Boxplots show the MCC distribution of the models from the 15 groups, including TSN, that predicted all original and swap endpoints from the MAQC-II. The original and swap MCC values are averaged for each group. In addition to endpoints A through I, a boxplot showing the mean MCC over endpoints A through H is shown (ALL). We exclude endpoint I from this final boxplot because it is a negative control. The bottom and top of each box indicate the lower and upper quartiles of the data, respectively. The middle line represents the median. The whiskers indicate the extreme values. The asterisk represents the performance of TSN on that dataset. All raw data is included in Additional file 3.

As stated above, only five of the six MAQC-II datasets are currently available from GEO, therefore we were only able to compare TSN to these datasets. All filtering and classification was performed using only the training data for each dataset – the validation set was left completely out of these calculations. Where possible (Affymetrix platforms), the features of each training set were first filtered for a high percentage (66%) of present or marginal calls using a MATLAB implementation of the Affymetrix MAS5 call algorithm [26]. The most differentially expressed probes for each training set were identified using the TSN implementation of the Wilcoxon rank sum test. Finally, the *dynamic N* TSN algorithm was used to identify the highest-scoring classifier on the training set over a range of *N* = {2,3,4} and DEG = {16,10,9}. As described in the methods section, the algorithm was allowed to select the best value of *N* using apparent accuracy of the training set. The highest scoring classifier was then applied to the validation set for each endpoint. The results of the TSN algorithm models applied to each endpoint validation set in the context of all analyzed participants are shown in Figure 5. All raw data is included in Additional file 3. The mean MCC value across all endpoints (excluding endpoint I, the negative control) was also calculated for each participant, and is shown in Figure 5. TSN performs competitively on these datasets, yielding a mean MCC value across all endpoints of 0.444. The maximum mean MCC value achieved by any of the groups was SAI, with 0.489.

In addition to standard cross validation and validation set MCC, we also measured the statistical significance of different classifier sizes. As described with the cancer datasets above, we ran 100 iterations of TSN using fixed values of *N* = 2, *N* = 3, and *N* = 4, as well as *dynamic N =* {2,3,4} on all nine of the MAQC-II training sets. For example, in endpoints A and B, *N* = 4 yields a statistically significant improvement over smaller classifier sizes. For endpoints C and E, *N =* 2 is the most effective classifier size. For endpoint G, there was no significant difference between any of the classifier sizes. In six out of the nine datasets (endpoints A, C, F, G, H, and I) there was no significant difference in MCC between *dynamic N* and the highest-scoring fixed value of *N*. The complete results are available in Additional file 1: Figure S5. All raw data is included in Additional file 4.

**Figure 6:**
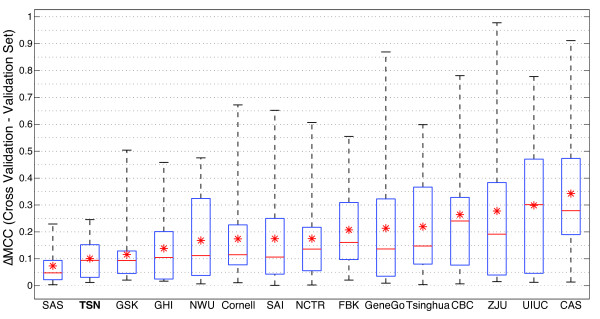
**ΔMCC Results from MAQC-II data.** Boxplots showing the distribution of **Δ**MCC values on the original data for each group, where **Δ**MCC = Cross Validation MCC – Validation Set MCC. This illustrates the amount of overfitting present during cross validation. The absolute value of each **Δ**MCC value was used in the calculations. The cross validation performed for TSN was 5-fold cross validation, repeated 10 times, as recommended by the MAQC-II consortium. Boxplots are sorted by the mean ΔMCC for each group (asterisk). All raw data is included in Additional file 3.

In order to test the amount of overfitting, we calculated the difference of the MCC values from each validation set and the corresponding MCC values from training set cross validation for each group. The cross validation performed for TSN was 5-fold cross validation, repeated 10 times, as recommended by the MAQC-II consortium. These results are presented in Figure 6 as boxplots showing the distribution of ΔMCC values. To prevent negative and positive values canceling each other out, the absolute value of each ΔMCC was used. Both original and swap datasets were included in the calculation of ΔMCC. TSN has a mean ΔMCC = 0.101, ranking second after SAS for the lowest ΔMCC of any of the MAQC-II participants – demonstrating that TSN had a remarkably low overfitting to the data.

For all analyses in this paper, up to sixteen differentially expressed genes were selected by the Wilcoxon rank sum test to input into the TSN algorithm. The fact that so few features were input to TSN in these analyses could explain the low levels of overfitting it exhibits. To test this, we ran all MAQC-II training sets (except for the negative control endpoint I, which would bias the results of ΔMCC towards zero) over a range of input feature sizes. For *N* = 2, we input a range of 16 to 10,000 input features. For *N* = 3 we input a range of 10 to 670 input features. For *N* = 4 we input a range of 9 to 188 input features. These numbers were chosen to span approximately the same range of possible feature combinations for each value of *N* (approximately 120 combinations up to 50 million combinations). Finally we ran *dynamic N* for *N =* {2,3,4} over the same ranges of input feature sizes. ΔMCC values were calculated for each input feature size, and box plots of their distributions are shown in Additional file 1: Figure S6. All raw data is included in Additional file 5. While the mean ΔMCC values do increase as a function of input feature size, overall the levels of overfitting remain low for TSN despite the increase. The mean ΔMCC exhibited by *dynamic N* TSN at the largest input size of [10000, 760, 188], is 0.148. This is still among the smallest mean ΔMCC value observed in any of the participating groups; only three groups are smaller (GHI, GSK, and SAS).

## Conclusions

The goal of relative expression classification algorithms is to identify simple yet effective classifiers that are resistant to data normalization procedures and overfitting, practical to implement in a clinical environment, and potentially biologically interpretable. The top-scoring ‘N’ algorithm presented here retains these desirable properties while allowing a larger combination and permutation space to be searched than that afforded by earlier relative expression algorithms such as TSP and TST. TSN can also recommend the classifier size (*N)* most likely to result in effective classification based on the training set. Of course, more care must be taken to avoid overfitting with TSN, particularly on smaller datasets, given that the permutation space grows with the factorial numbers. However, the problem of overfitting can be well mitigated by choosing a suitably small number of features from which to build the classifier, or ensuring that the number of samples available is large enough to justify searching a larger combination space. All the results presented in this paper were performed using between nine and sixteen features of the microarray datasets. TSN is therefore well suited for datasets of emerging technologies that contain smaller numbers of features to begin with, such as secretomics and miRNAs. However, as Figure 3 demonstrates, it is still possible to search tens of thousands of permutations in a relatively short amount of time, when justified by large sample sizes. The statistical significance of the resulting classifiers can then be determined though e.g. permutation tests of the class labels.

We have demonstrated the effectiveness of TSN in classification of the MAQC-II datasets in comparison with many other classification strategies, including artificial neural networks, classification trees, discriminant analysis, k-Nearest neighbor, naïve Bayes, and support vector machines, as implemented by several universities and companies from around the world. We do not claim that TSN is necessarily the best or most effective classifier for every circumstance. For example, TSN performs relatively poorly on endpoint H, which as the positive control in which classes were simply assigned as the gender of the study participants, should be among the easiest to classify. A major strength of the algorithm is the level to which the MCC values for cross validation agree with the MCC values on the independent validation set (ΔMCC). Importantly, these results indicate a very low level of overfitting, and increase our confidence that results generated through cross validation on future datasets will be effective classifiers on independent validation sets. That is, when TSN works on a dataset it is relatively more likely to be true, and conversely, when it is going to fall short in independent validation it typically does not work well in cross validation and so can be discarded as a candidate diagnostic early in the process. Analyses over a range of input sizes indicate that overfitting remains low even as input feature numbers increase, given sufficient sample sizes.

Of all the MAQC-II participants, including TSN, group SAS yielded the lowest mean ΔMCC score (0.074), indicating low levels of overfitting. Group SAI yielded the highest mean MCC (0.4893) for original and swap datasets, indicating high levels of validation set accuracy based on the training set. Both of these groups utilized multiple classification strategies across all endpoints. For example, group SAS used logistic regression for endpoints A, E, and I, support vector machines for endpoints B, G, and H, partial least squares regression for endpoints D and F, and a decision tree for endpoint C. Group SAI used support vector machines for endpoints A, B, E, F, G, and I, k-nearest neighbor for endpoints C and H, and a machine learning classifier for endpoint D. Group SAI also used a range of different feature selection methods for each endpoint. Both groups also used different classification strategies for the swap datasets. For example, group SAS used logistic regression for the original endpoint E data but partial least squares regression on swap endpoint E. Group SAI used a machine learning classifier for the original endpoint D, and discriminant analysis for swap endpoint D [11]. As a result, TSN is not only being compared to different classification strategies, but an ensemble of classification strategies that were chosen in an attempt to maximize success for each endpoint across both original and swap datasets. Given its advantages of relative simplicity, biological interpretability, and low levels of overfitting, the TSN algorithm can serve as a useful tool for hypothesis generation, particularly as next generation sequencing and proteomics technologies yield increasing sensitivity in biomolecule measurements.

## Abbreviations

CPU: Central processing unit; DEG: Differentially expressed genes; GPU: Graphics processing unit; MAQC-II: Microarray quality control II; MCC: Matthews correlation coefficient; TSN: Top-scoring ‘N’; TSP: Top-scoring pair; TST: Top-scoring triplet.

## Competing interests

The authors declare that they have no competing interests.

## Authors' contributions

AM conceived of the study, wrote the software, and drafted the manuscript. NP participated in the study design and helped to write the manuscript. All authors read and approved the final manuscript.

## Supplementary Material

Additional file 1 **Figure S1. Counting systems. Figure S2: Three complete conversions from permutation to decimal. Figure S3: Pseudocode for the core operation of the TSN algorithm on the GPU. Figure S4: Cancer dataset statistical tests for differences between values of *N*. Figure S5: MAQC-II Statistical tests for differences between values of *N*. Figure S6: ΔMCC box plots for different input sizes on the MAQC-II dataset. ** Click here for file

Additional file 2 **Raw data for statistical significance testing of cancer results referenced in Figure **4**.** Click here for file

Additional file 3 **Raw data for cross validation and test set MCC scores and ΔMCC scores for all MAQC-II participants and TSN, referenced in Figures **5** and **6**.** Click here for file

Additional file 4 Raw data for statistical significance testing of MAQC-II results referenced in Figure S5. Click here for file

Additional file 5 Raw data for ΔMCC values over a range of input feature sizes referenced in Figure S6. Click here for file
