# Supplementary material for: The top-scoring ‘N’ algorithm: a generalized relative expression classification method from small numbers of biomolecules
Source: BMC Bioinformatics. 2012 Sep 11;13:227. doi: 10.1186/1471-2105-13-227 (PMC3663421; doi:10.1186/1471-2105-13-227)
Supplement: Additional file 1 — Figure S1. Counting systems. Figure S2: Three complete conversions from permutation to decimal. Figure S3: Pseudocode for the core operation of the TSN algorithm on the GPU. Figure S4: Cancer dataset statistical tests for differences between values of N . Figure S5: MAQC-II Statistical tests for differences between values of N . Figure S6: ΔMCC box plots for different input sizes on the MAQC-II dataset. [file 1471-2105-13-227-S1.pdf]

The Top-Scoring ‘N’ Algorithm: A Generalized Relative Expression Classification Method from Small Numbers of Biomolecules

Andrew T. Magis[1,2] and Nathan D. Price[1,2]

[1]Institute for Systems Biology, Seattle, WA

[2]Center for Biophysics and Computational Biology, University of Illinois, Urbana, IL

SUPPLEMENTAL SECTION

Decimal Counting System (Fixed radix)

Place	10^5	10^4	10^3	10^2	10^1	10^0
Max Digit	9	9	9	9	9	9
Digit	0	0	0	0	4	2

$4x10^1 + 2x10^0 = \mathbf{42}$

Binary Counting System (Fixed radix)

Place	2^5	2^4	2^3	2^2	2^1	2^0
Max Digit	1	1	1	1	1	1
Digit	1	0	1	0	1	0

$1x2^5 + 0x2^4 + 1x2^3 + 0x2^2 + 1x2^1 + 0x2^0 = \mathbf{42}$

Factorial Counting System (Mixed radix)

Place	5!	4!	3!	2!	1!	0!
Max Digit	5	4	3	2	1	0
Digit	0	1	3	0	0	0

$1x4! + 3x3! + 0x2! + 0x1! + 0x0! = \mathbf{42}$

Figure S1: Counting systems

Representation of a number (42) in three different counting systems: decimal (42), binary (101010), and factorial (13000). Note that decimal and binary are both fixed radix systems, in which the multiplicative distance between each digit place is the same, and the set of digits used for each place is the same. The factorial counting system (factoradics) uses a mixed-radix system in which the multiplicative distance between each digit place is not the same, and the set of digits used for each place is also not the same.

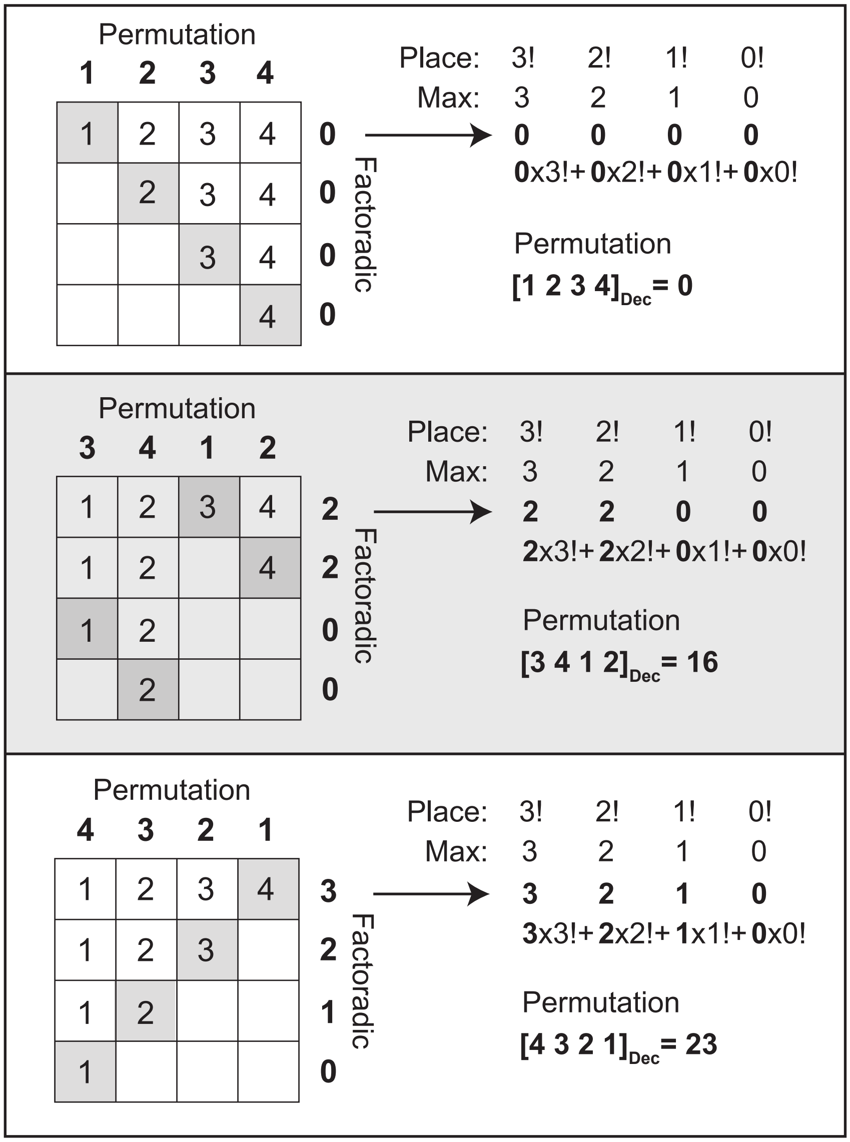

Figure S2: Three complete conversions from permutation to decimal

Three complete translations from permutation to decimal, by way of the factoradic, for a set of size 4. The permutation to be translated is shown above the grid box, and the factoradic representation of that permutation is listed to the right of the grid box. Each row of the grid box determines a digit of the factoradic. The first row of the grid box is the complete sorted list. To perform the translation, consider the elements of the permutation progressively from 1 to 4. The digit of the factoradic is equal to the number of elements of the sorted list to the left of this position. After each digit of the permutation is considered, the corresponding digit of the sorted list is removed. Note that the sorted permutation (**Top**) translates into decimal representation 0. The reverse sorted permutation (**Bottom**) translates into decimal representation 4! - 1 = 23, the maximum number that can be represented by a factoradic of size 4.

```
 1. INPUTS: C1,C2 = Ranked feature expression data
 2.            M = num features of C1,C2
 3.            N = classifier size
 4. copy C1,C2 to GPU
 5. hist1,hist2 = allocate zeroed-out N! size histograms on GPU
 6. num_combinations = (M choose N)
 7. num_iterations = num_combinations / GPU memory
 8. foreach iteration in num_iterations (CPU)
 9.     combs = combinations for this iteration
10.     copy combs to GPU
11.     parallel foreach combination C in combs (GPU)
12.         foreach sample S in C1
13.             fact = factoradic for permutation of C in S
14.             hist1[decimal(fact)] = hist1[decimal(fact)] + 1
15.         foreach sample S in C2
16.             fact = factoradic for permutation of C in S
17.             hist2[decimal(fact)] = hist2[decimal(fact)] + 1
18.         hist1 = normalize(hist1);  hist2 = normalize(hist2)
19.         scores = sum(hist1 - hist2)
20.     copy scores,hist1,hist2 to CPU
21.     sort scores and save (CPU)
22. classifiers = top combs over all iterations
23. OUTPUTS: top classifiers, scores, hist1, hist2
```

Figure S3: Pseudocode for the core operation of the TSN algorithm on the GPU.

This pseudocode does not include code to choose the value of N using apparent accuracy or determine classification accuracy using cross validation. Lines 11 through 19 are computed in parallel on the GPU. The GPU is called multiple times depending on the number of combinations requested and the size of the GPU device memory.

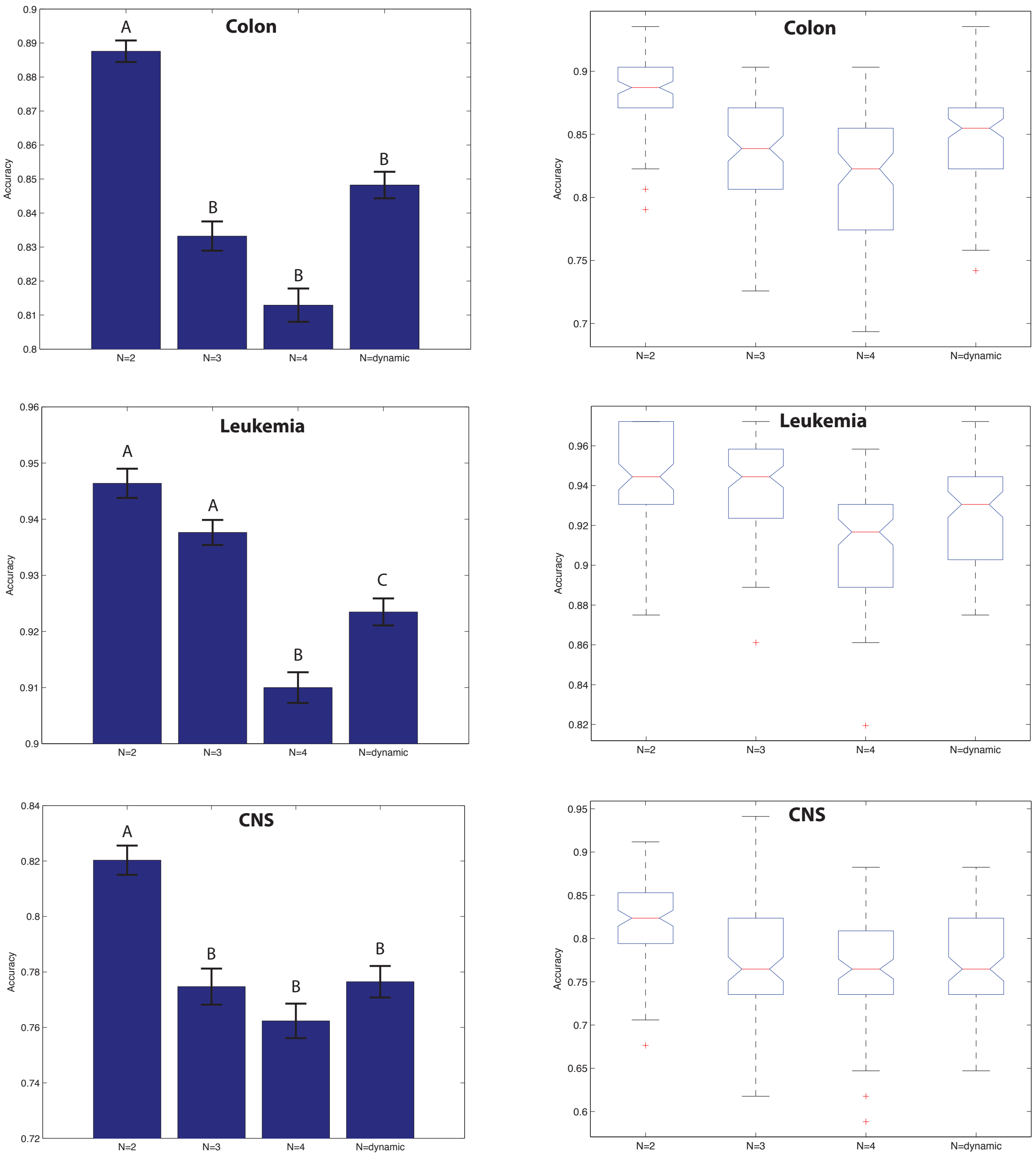
Colon
Accuracy
A
B
B
B
N=2
N=3
N=4
N=dynamic
Colon
Accuracy
N=2
N=3
N=4
N=dynamic
Leukemia
Accuracy
A
A
B
C
N=2
N=3
N=4
N=dynamic
Leukemia
Accuracy
N=2
N=3
N=4
N=dynamic
CNS
Accuracy
A
B
B
B
N=2
N=3
N=4
N=dynamic
CNS
Accuracy
N=2
N=3
N=4
N=dynamic

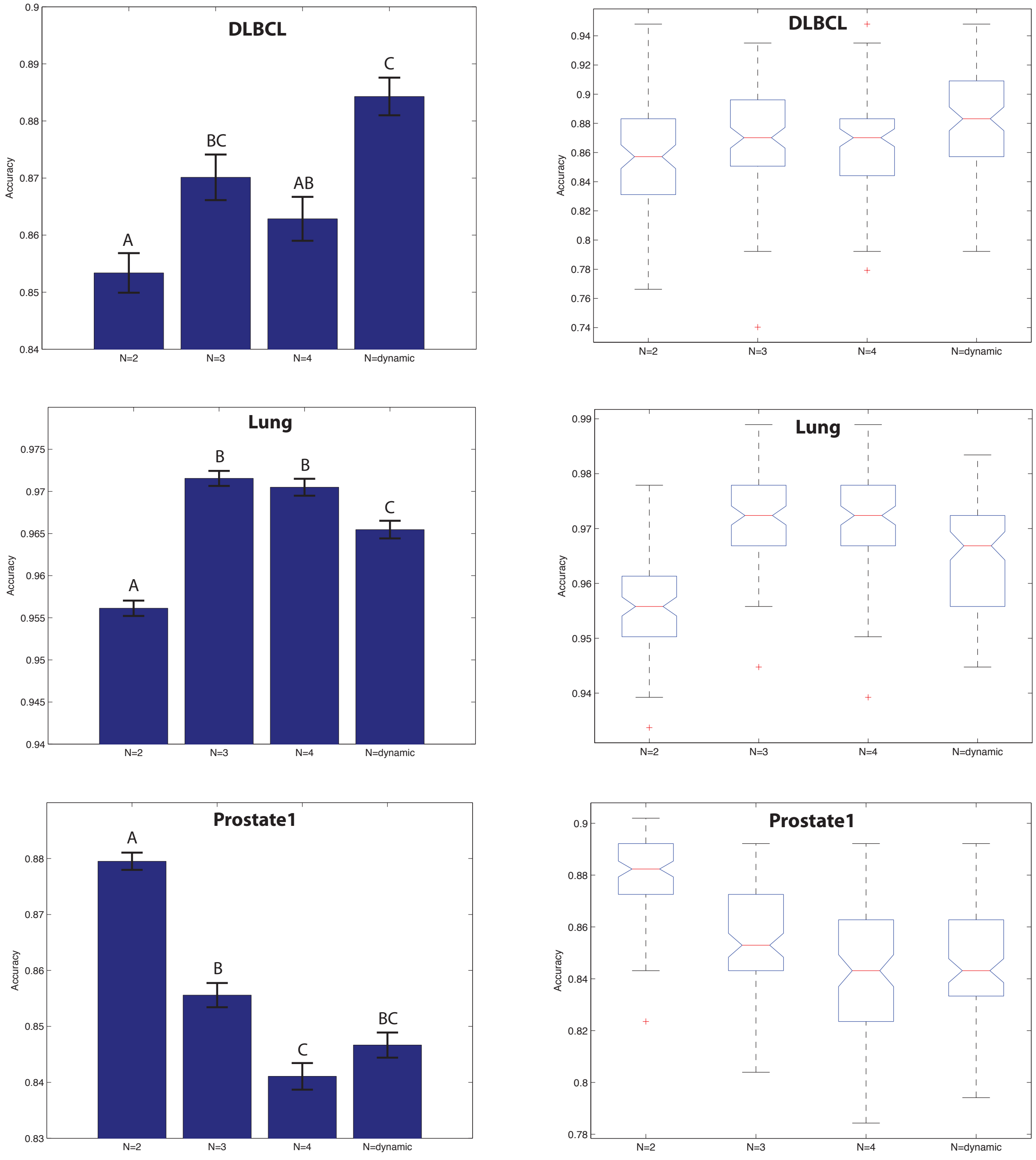
DLBCL
Lung
Prostate1
Accuracy
N=2
N=3
N=4
N=dynamic
A
B
C
AB
BC
0.84
0.85
0.86
0.87
0.88
0.89
0.9
0.74
0.76
0.78
0.8
0.82
0.92
0.94
0.94
0.945
0.95
0.955
0.96
0.965
0.97
0.975
0.98
0.99
0.83

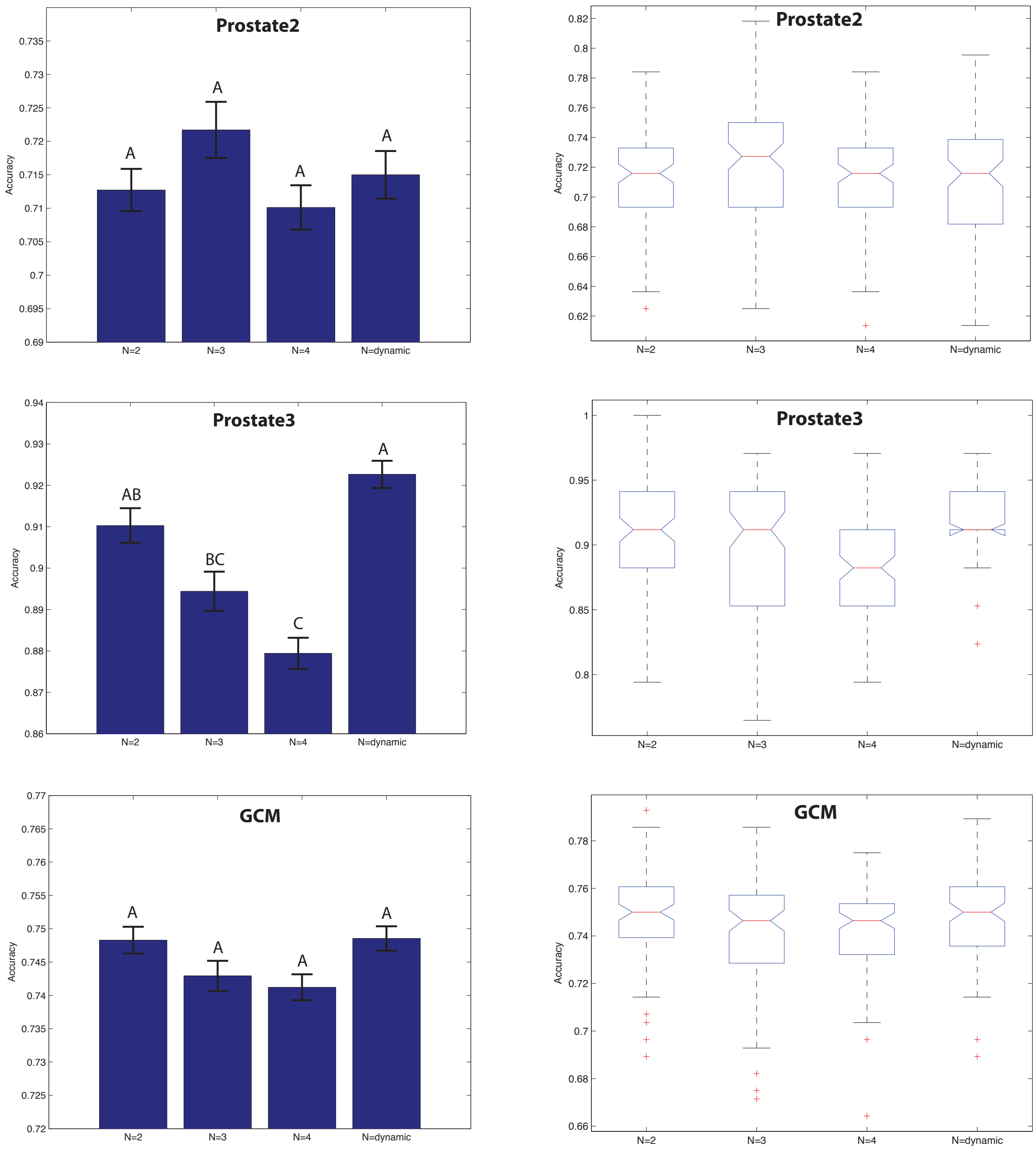

Figure S4: Cancer dataset statistical tests for differences between values of *N*.

100 iterations of 5-fold cross validation were run using TSN on all nine cancer datasets cited in the paper. Four different types of TSN were run: *N*=2, *N*=3, *N*=4, and *dynamic N*, where the algorithm was allowed to choose the value of *N* at each iteration of the cross validation loop. These four groups were tested using the nonparametric Kruskal-Wallis test to determine significant differences between the accuracies. A p-value < 0.05 was considered significant. Error bars in the bar plot indicate standard error, not confidence

intervals. Letters above each bar indicate membership in a significance group. If two bars share the same letter, they are not significantly different. If two bars do not share the same letter, they are significantly different. All raw data is included in Additional file 2.

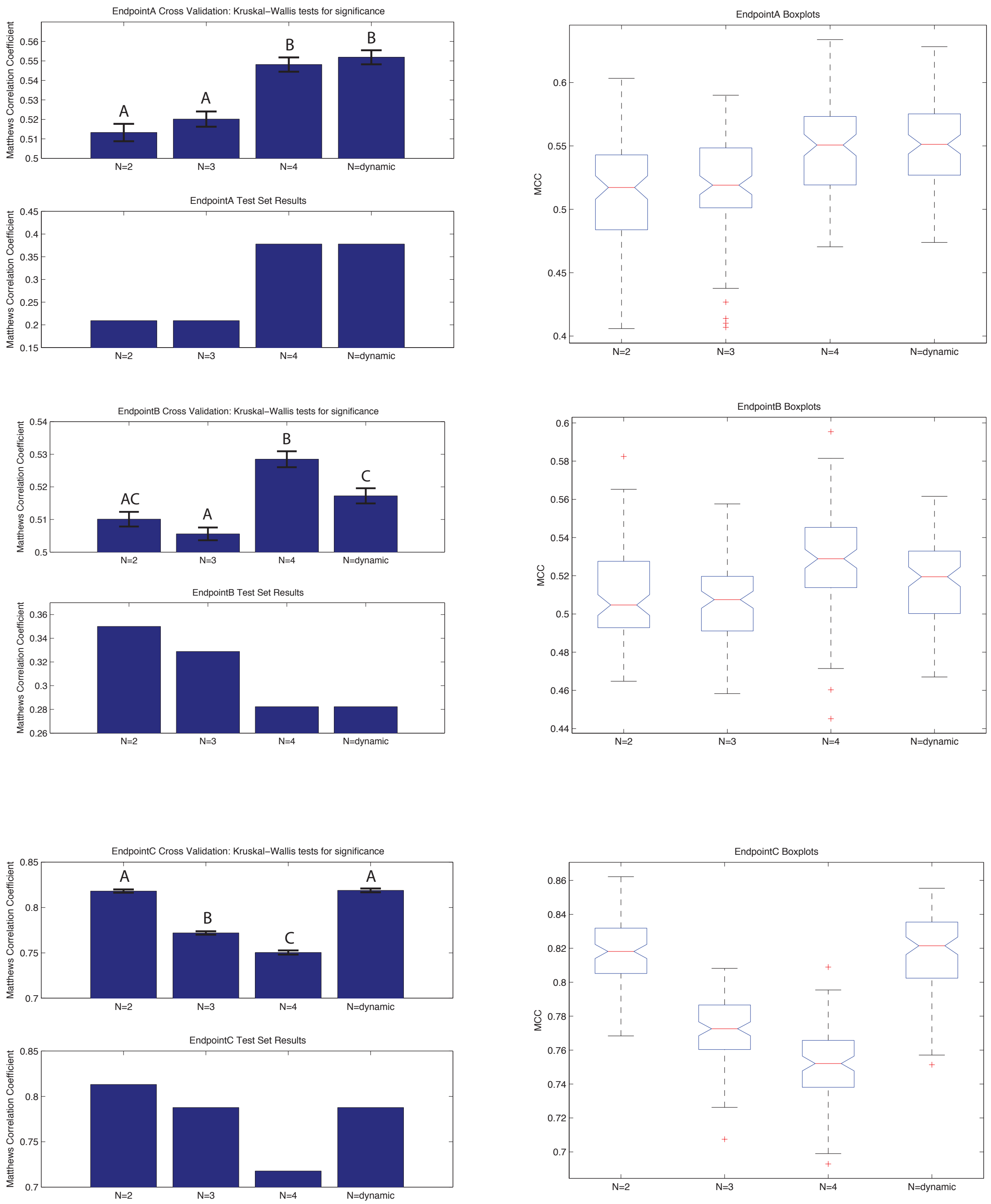
EndpointA Cross Validation: Kruskal–Wallis tests for significance
Matthews Correlation Coefficient
0.5 0.51 0.52 0.53 0.54 0.55 0.56
A A B B
N=2 N=3 N=4 N=dynamic
EndpointA Test Set Results
Matthews Correlation Coefficient
0.15 0.2 0.25 0.3 0.35 0.4 0.45
N=2 N=3 N=4 N=dynamic
EndpointA Boxplots
MCC
0.4 0.45 0.5 0.55 0.6
N=2 N=3 N=4 N=dynamic
EndpointB Cross Validation: Kruskal–Wallis tests for significance
Matthews Correlation Coefficient
0.5 0.51 0.52 0.53 0.54
AC A B C
N=2 N=3 N=4 N=dynamic
EndpointB Test Set Results
Matthews Correlation Coefficient
0.26 0.28 0.3 0.32 0.34 0.36
N=2 N=3 N=4 N=dynamic
EndpointB Boxplots
MCC
0.44 0.46 0.48 0.5 0.52 0.54 0.56 0.58 0.6
N=2 N=3 N=4 N=dynamic
EndpointC Cross Validation: Kruskal–Wallis tests for significance
Matthews Correlation Coefficient
0.7 0.75 0.8 0.85
A B C A
N=2 N=3 N=4 N=dynamic
EndpointC Test Set Results
Matthews Correlation Coefficient
0.7 0.75 0.8 0.85
N=2 N=3 N=4 N=dynamic
EndpointC Boxplots
MCC
0.7 0.72 0.74 0.76 0.78 0.8 0.82 0.84 0.86
N=2 N=3 N=4 N=dynamic

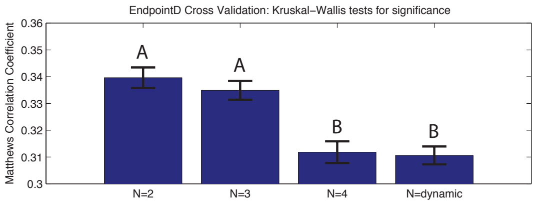
EndpointD Cross Validation: Kruskal–Wallis tests for significance
Matthews Correlation Coefficient
A
A
B
B
N=2
N=3
N=4
N=dynamic

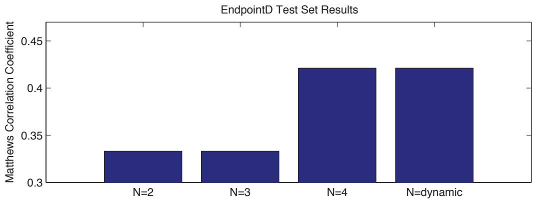
EndpointD Test Set Results
Matthews Correlation Coefficient
N=2
N=3
N=4
N=dynamic

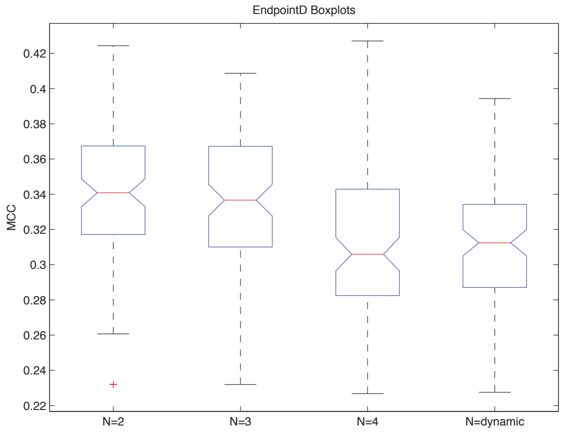
EndpointD Boxplots
MCC
N=2
N=3
N=4
N=dynamic

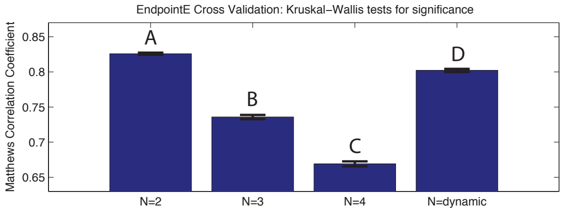
EndpointE Cross Validation: Kruskal–Wallis tests for significance
Matthews Correlation Coefficient
A
B
C
D
N=2
N=3
N=4
N=dynamic

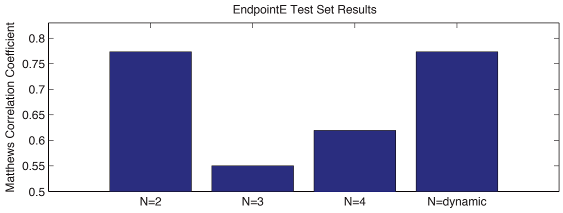
EndpointE Test Set Results
Matthews Correlation Coefficient
N=2
N=3
N=4
N=dynamic

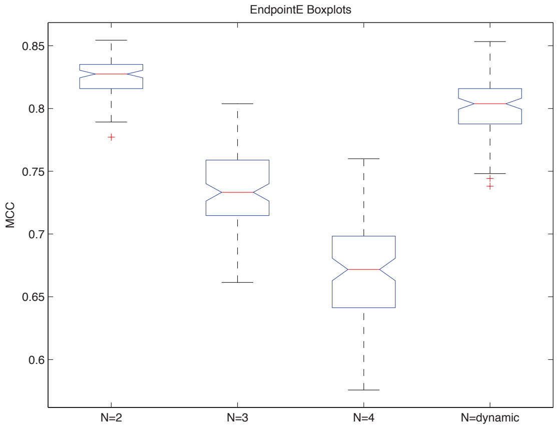
EndpointE Boxplots
MCC
N=2
N=3
N=4
N=dynamic

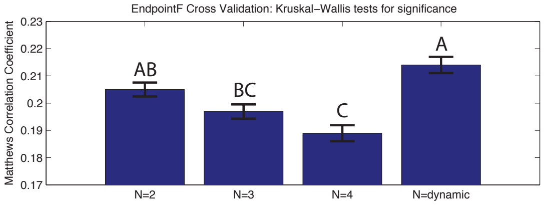
EndpointF Cross Validation: Kruskal–Wallis tests for significance
Matthews Correlation Coefficient
AB
BC
C
A
N=2
N=3
N=4
N=dynamic

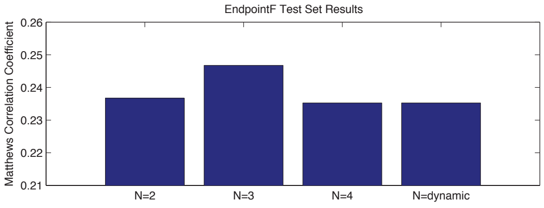
EndpointF Test Set Results
Matthews Correlation Coefficient
N=2
N=3
N=4
N=dynamic

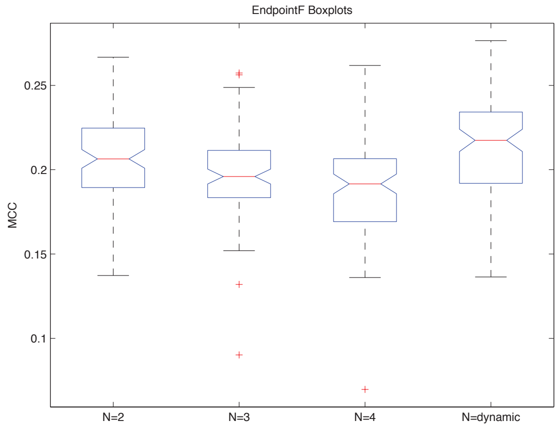
EndpointF Boxplots
MCC
N=2
N=3
N=4
N=dynamic

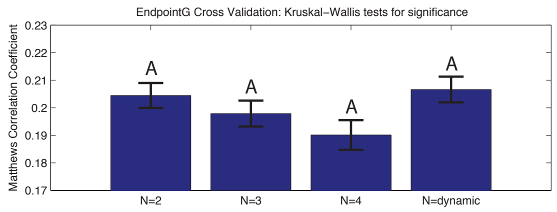
EndpointG Cross Validation: Kruskal−Wallis tests for significance
Matthews Correlation Coefficient
0.23
0.22
0.21
0.2
0.19
0.18
0.17
A
A
A
A
N=2
N=3
N=4
N=dynamic

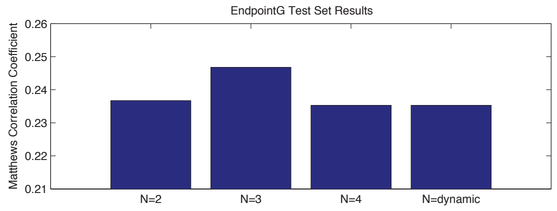
EndpointG Test Set Results
Matthews Correlation Coefficient
0.26
0.25
0.24
0.23
0.22
0.21
N=2
N=3
N=4
N=dynamic

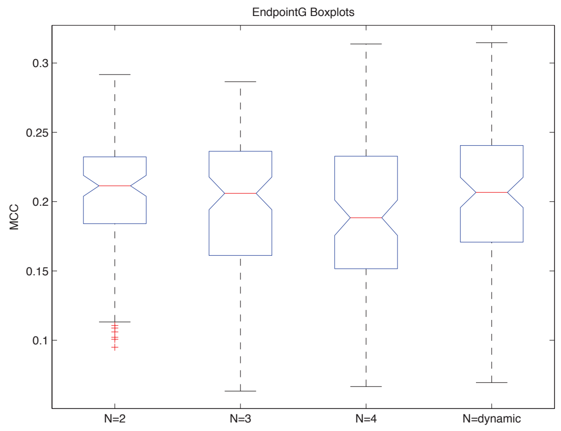
EndpointG Boxplots
MCC
0.3
0.25
0.2
0.15
0.1
N=2
N=3
N=4
N=dynamic

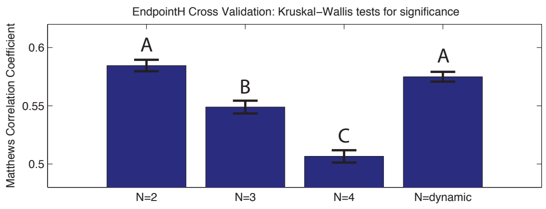
EndpointH Cross Validation: Kruskal−Wallis tests for significance
Matthews Correlation Coefficient
0.6
0.55
0.5
A
B
C
A
N=2
N=3
N=4
N=dynamic

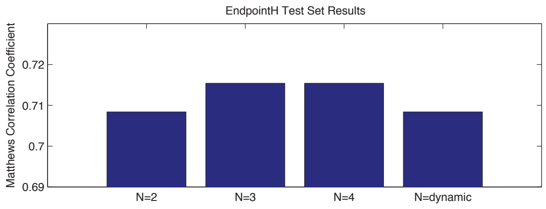
EndpointH Test Set Results
Matthews Correlation Coefficient
0.72
0.71
0.7
0.69
N=2
N=3
N=4
N=dynamic

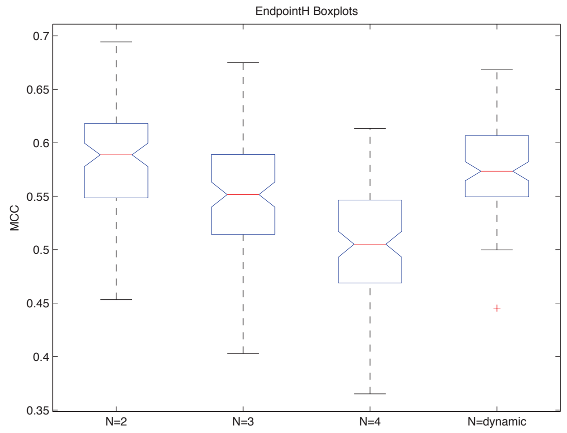
EndpointH Boxplots
MCC
0.7
0.65
0.6
0.55
0.5
0.45
0.4
0.35
N=2
N=3
N=4
N=dynamic

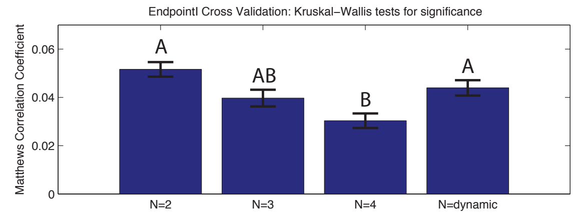
EndpointI Cross Validation: Kruskal−Wallis tests for significance
Matthews Correlation Coefficient
0.06
0.04
0.02
0
A
AB
B
A
N=2
N=3
N=4
N=dynamic

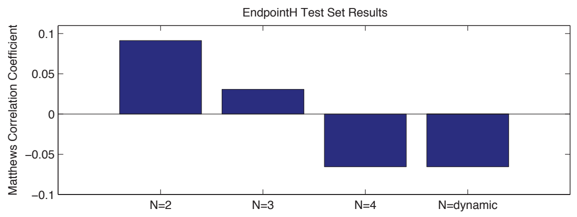
EndpointH Test Set Results
Matthews Correlation Coefficient
0.1
0.05
0
−0.05
−0.1
N=2
N=3
N=4
N=dynamic

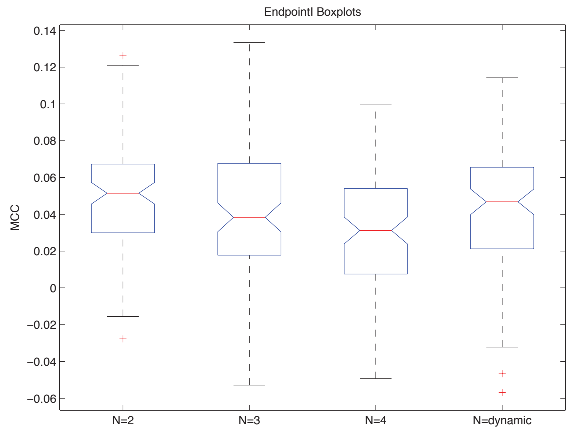
EndpointI Boxplots
MCC
0.14
0.12
0.1
0.08
0.06
0.04
0.02
0
−0.02
−0.04
−0.06
N=2
N=3
N=4
N=dynamic

Figure S5: MAQC-II Statistical tests for differences between values of *N*.

100 iterations of 5-fold cross validation were run using TSN on all nine MAQC-II datasets cited in the paper. Four different types of TSN were run: *N*=2, *N*=3, *N*=4, and *dynamic N*, where the algorithm was allowed to choose the value of *N* at each iteration of the cross validation loop. These four groups were tested using the nonparametric Kruskal-Wallis test to determine significant differences between the accuracies. A p-value < 0.05 was considered significant. Error bars in the bar plot indicate standard error, not confidence intervals. Letters above each bar indicate membership in a significance group. If two bars share the same letter, they are not significantly different. If two bars do not share the same letter, they are significantly different. All raw data is included in Additional file 4.

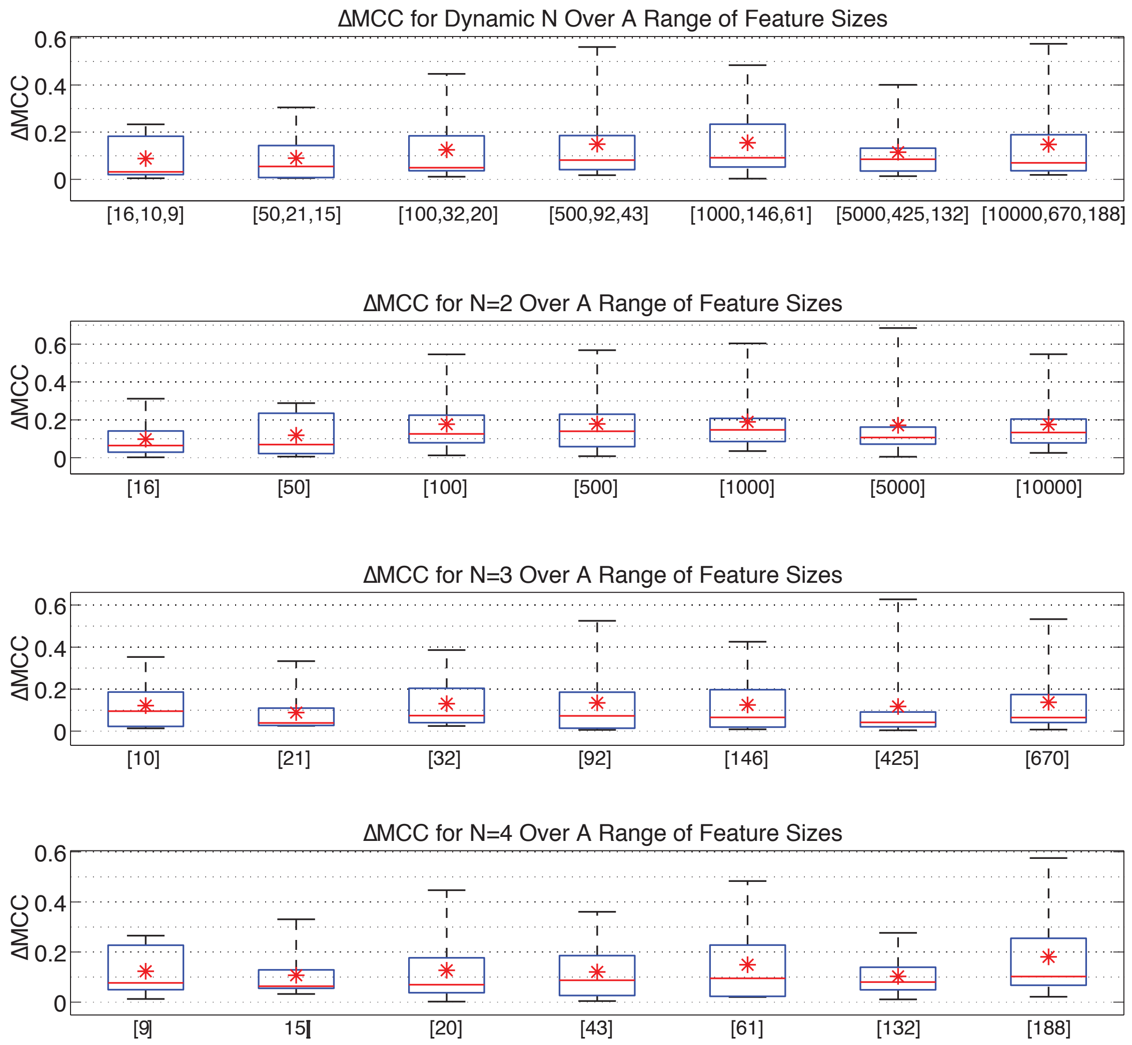

Figure S6: ΔMCC boxplots for different input sizes on the MAQC-II dataset

The TSN algorithm is tested in the paper with very small input sizes (up to 16 features). When the cross validation MCC is compared with the test set MCC, the average difference is very small (Figure 6), indicating that the level of overfitting with the TSN algorithm is low. To determine if the low overfitting of the TSN algorithm is due to the small number of input features we tested all the MAQC-II datasets again using a large range of input sizes. This process was performed for fixed values of *N*=2, *N*=3, and *N*=4, as well as *dynamic N*. The number of features was chosen to yield approximately the same number of combinations for each classifier size. The feature sizes were chosen to span a range of combinations from 120 to 50,000,000. The top panel shows the distribution of ΔMCC scores for *dynamic N*, and the 2nd, 3rd, and 4th panels show the same calculations for fixed *N*=2, *N*=3, and *N*=4, respectively. Despite the wide range of input feature sizes, the mean (asterisk) and median (middle line) ΔMCC scores stay low, among the lowest of any of the MAQC-II participants. The distance between the lower and upper quartiles of the data also remains fairly constant, as indicated by the top and

bottom of the box. The whiskers indicate the extreme values. All raw data is included in Additional file 5.
